# The 9-item Concise Health Risk Tracking – Self-Report (CHRT-SR_9_) measure of suicidal risk: Performance in adult primary care patients

**DOI:** 10.3389/fpsyt.2023.1014766

**Published:** 2023-02-14

**Authors:** Karabi Nandy, A. John Rush, Thomas J. Carmody, Taryn L. Mayes, Madhukar H. Trivedi

**Affiliations:** ^1^Peter O’Donnell School of Public Health, The University of Texas Southwestern Medical Center, Dallas, TX, United States; ^2^Department of Psychiatry, The University of Texas Southwestern Medical Center, Dallas, TX, United States; ^3^Curbstone Consultant LLC., Santa Fe, NM, United States; ^4^Department of Psychiatry and Behavioral Sciences, Duke University School of Medicine, Durham, NC, United States; ^5^Department of Psychiatry, Duke-National University of Singapore, Singapore, Singapore

**Keywords:** psychometrics, concise health risk tracking scale self-report (CHRT-SR), adults, depression, suicidal risk, suicidality

## Abstract

**Purpose:**

To evaluate the psychometric properties of a 9-item Concise Health Risk Tracking Self-Report (or CHRT-SR_9_) to assess suicidal risk in adult primary care outpatients.

**Methods:**

Overall, 369 adults completed the original 14-item version of CHRT-SR at baseline and within 4 months thereafter, from which the CHRT-SR_9_ was extracted using multigroup confirmatory factor analysis. Measurement invariance (across age and sex) and classical test theory characteristics of the CHRT-SR_9_ were evaluated. Concurrent validity was assessed by comparing CHRT-SR_9_ responses to those of the suicide item in the Patient Health Questionnaire (PHQ-9), both cross-sectionally and as a change measure over time.

**Results:**

Confirmatory factor analysis identified the CHRT-SR_9_ as the optimal solution. Factors included pessimism, helplessness, despair (2 items each) and suicidal thoughts (3 items). Measurement invariance held across sex and age groups, indicating that mean differences among sub-groups were real and not attributable to measurement bias. Classical test theory revealed acceptable item-total correlations overall (0.57–0.79) and internal consistency (Spearman–Brown from 0.76 to 0.90). Concurrent validity analyses revealed that the CHRT-SR_9_ can measure both improvement and worsening of suicidality over time. A PHQ-9 response of 0, 1, 2, and 3 on the suicide item corresponded to 7.82 (5.53), 16.80 (4.99), 20.71 (5.36), and 25.95 (7.30) (mean and SD) on CHRT-SR_9_ total score, respectively.

**Conclusion:**

The CHRT-SR_9_ is a brief self-report evaluating suicidality with excellent psychometric properties that is sensitive to change over time.

## Introduction

Suicide is a significant public health crisis in the United States, with one of the highest rates of suicide among wealthy countries (1). In the US, the annual suicide rate increased 30% between 2000 and 2020, from 10.4 suicides per 100,000 to 13.5/100,000 (2).

A large longitudinal study found that 83% of persons who died by suicide received healthcare services in the year before their death, and 50% received them within the prior month (3). These findings prompted regulatory agencies and healthcare organizations to develop guidelines for physicians to routinely screen patients for depressive symptoms. Screening for risk of suicide, however, was reserved only for those who screened positive for depression or substance abuse (4). There is much debate about whether to extend suicide risk screening to all patients in the primary care setting. Some have pointed out that suicidality can occur even in the absence of major risk factors like depression (5). Sentinel event alert 56 (2016) recommended that physicians in primary care setting screen all patients for suicidal ideation (6). They advised using a brief, standardized, evidence-based screening tool.

**FIGURE 1:**
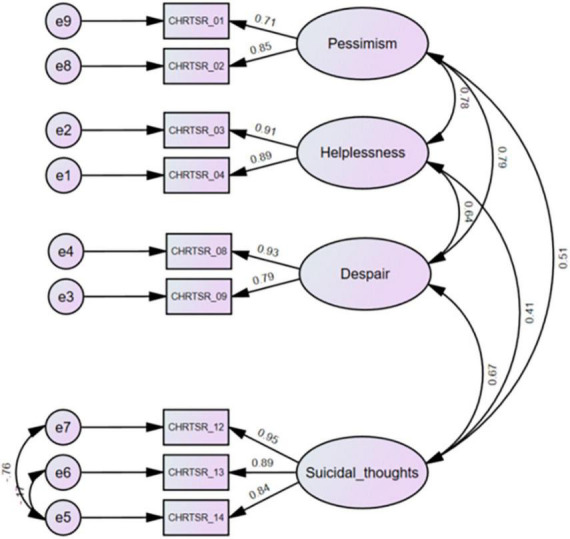
Confirmatory factor analysis of the CHRT-SR_9_ scale at the first visit (*n* = 369). CHRTSR_01: I feel as if things are never going to get better; CHRTSR_02: I have no future; CHRTSR_03: It seems as if I can do nothing right; CHRTSR_04: Everything I do turns out wrong; CHRTSR_08: I feel that there is no reason to live; CHRTSR_09: I wish I could just go to sleep and not wake up; CHRTSR_12: I have been having thoughts of killing myself; CHRTSR_13: I have thoughts about how I might kill myself; CHRTSR_14: I have a plan to kill myself.

There exist a variety of measurement tools that study behaviors related to suicide risk (7). These rating scales typically include the categories of assessment measures such as suicidal ideation and behavior, lethality of suicide attempts, reasoning mechanisms of suicide attempters, etc. This report evaluates a shortened version of the 14-item Concise Health Risk Tracking Self Report (CHRT-SR) (8). The original version included constructs such as pessimism, helplessness, social support, despair, impulsivity, and suicidal thoughts, measured on 5-point likert scales from “0: Strongly Disagree” to “4: Strongly Agree.” Recent work in a representative sample of adolescent outpatients revealed that a 9-item scale was psychometrically the best and most preferred of the various versions (9). These 9 items include items measuring *pessimism* (items 1 and 2), *helplessness* (items 3 and 4), *despair* (items 8 and 9), and *current suicidal thought and plans* (items 12, 13, and 14) from the 14-item CHRT-SR (Figure 1). This report extends work on the 9-item version of the CHRT-SR (or CHRT-SR_9_) to evaluate its performance with adult outpatients in primary care. Establishing that the CHRT-SR_9_ performs satisfactorily in this adult primary care setting would be a significant step in developing evidence of the scale’s reliability and sensitivity to change across a wide age range seen in various settings.

This report aimed to:

(1) Conduct confirmatory factor analysis of CHRT-SR_9_ in a representative sample of adults,
(2) Assess the measurement invariance of CHRT-SR_9_ by sex and age-groups,
(3) Assess the classical test theory (CTT) psychometrics of CHRT-SR_9_,
(4) Assess its performance against the suicide item of another independent scale (the major depressive disorder module of the Patient Health Questionnaire, PHQ-9) both cross-sectional and as a measure of change over time.

## Materials and methods

### Study design and participants

Data used in this report came from a joint quality improvement project of UT Southwestern Medical Center and primary care and specialty care clinics designed to facilitate and enhance access to evidence-based screening and treatment of depression (10). The initiative mandates that participating clinics conduct yearly depression screenings on every patient. Patients fill out a condensed, two-item version of the Patient Health Questionnaire (PHQ-2) on the first screen of the iPad application. If a patient’s PHQ-2 result is positive, indicative of depression, then the application will automatically administer other tests, such as the complete PHQ-9. The application shows these findings to healthcare professionals and helps them choose a diagnosis and a course of treatment. Data collection began in 2014 and is still ongoing. The UT Southwestern Medical Center Institutional Review Board approved this study with a waiver of the need to obtain informed consent from individual patients.

The project included a depression screening using the first 2 items of the PHQ-9. Patients who screened positive were given the full PHQ-9, the 14-item CHRT-SR, and a variety of other ratings scales measuring factors associated with depression (10). For this report, we focused on adults (≥18 years of age) who completed the CHRT-SR on two successive clinic visits, second visit being within 4 months of the first to approximate the time for a treatment trial and to minimize time differences between the two visits. Our analyzable sample included 369 adult patients in mostly primary care clinics (18 primary care and two specialty, cancer and obstetrics and gynecology).

### Measures

Demographic factors included sex (male/female), race (White, Black, Other) and age, based on self-report.

Concise Health Risk Tracking Scale Self-Report (CHRT-SR_14_): The 14-item CHRT-SR was designed to assess psychosocial and behavioral factors associated with increased risk of suicidal thoughts and behaviors. The items were designed as self-referent statements which respondents rated on 5-point scales starting at strongly disagree (0) to strongly agree (4), with higher scores indicative of greater severity of suicidality.

PHQ-9 is a nine-item, self-report inventory including all nine criterion symptoms that define a major depressive episode (11). Item 9 (“Thoughts that you would be better off dead, or of hurting yourself”) relates to suicidality/ideation. Each item is rated on a 4-point scale starting with “Not at all” (0) to “Nearly every day” (3), with higher scores indicating greater depression severity. Studies comprising eight primary care and seven obstetrical clinics demonstrated the diagnostic validity of the nine-item PHQ-9. Major Depressive Disorder was detected with 88% sensitivity and 88% specificity using PHQ-9 values greater than 10. The tool’s reliability and validity have shown that it possesses excellent psychometric features (11).

### Statistical data analyses

Confirmatory Factor Analysis (CFA) was conducted using AMOS 28 (12) to determine whether the 9-item CHRT-SR_9_ fits well to the CHRT-SR data in adults. We used maximum likelihood estimation to estimate model parameters and standard errors. Model-fit indices such as chi-square test, Comparative Fit Index (CFI), Tucker Lewis index (TLI), and Root-Mean-Square Error of Approximation (RMSEA) were investigated to assess model fit. Good fit thresholds for these indices are CFI > 0.95, TLI > 0.95, and RMSEA < 0.05 (13). Bollen–Stine bootstrap p was used as an indicator of model fit, since it operates without normality assumptions and *p* > 0.05 indicates excellent fit (14).

To assess whether CHRT-SR_9_ measured the same constructs across all respondents and demographic sub-groups, we evaluated measurement invariance by sex (male and female) and age-groups (young: 18–35, middle aged: 36–55, and older: >55 years). Measurement invariance can be categorized into three hierarchical levels, namely, configural (where the factor structure is the same across groups), metric (where factor loadings are similar across groups) and scalar (where values/means are also equivalent across groups) (15). We first tested whether the CFA fit for each sub-group separately (16). Thereafter, we tested for evidence of configural, metric and scalar invariance.

We calculated the Spearman–Brown coefficient to examine the internal consistency of the CHRT-SR_9_ using data from both the first and second visits. While Cronbach’s alpha is a popular measure of internal consistency, for a two-item scale, it usually underestimates the true reliability. On the other hand, the Spearman–Brown coefficient is less biased on average, especially if the correlation between the items is relatively strong (17).

We assessed its sensitivity to change over time by testing whether total and subscale means were different between first and second visits using paired sample *t*-tests.

We assessed its performance against Item 9 (the suicidal item) of the PHQ-9 as an anchor by testing whether means for each item, total score, and all subscale scores varied across response levels (0–3) to Item 9 of the PHQ-9 at the first visit.

Finally, we assessed whether CHRT-SR_9_ changed over time when individuals experienced a change in suicidality by looking at the mean change in response for each CHRT-SR_9_ item (as well as mean of total and subscale scores) against change in the PHQ-9 Item 9 over time (between second and first visits).

## Results

### Sample characteristics

Table 1 summarizes the sample characteristics. The majority were Whites (68.42%) and female (74.53%). The mean total score was 15.69 ± 8.14 for the CHRT-SR_9_ and 15.65 ± 6.77 for the PHQ-9.

**TABLE 1:** Baseline characteristics of the sample (*N* = 369).

	Mean	SD
Age (years)	36.44	15.41
**Age-groups**	***n* (%)**	
Young adults (18–35)	191 (51.76)	
Middle age (36–55)	116 (31.44)	
Older adulthood (>55)	62 (16.80)	
Males	94 (25.47%)	
**Race**		
White (%)	208 (68.42%)	
Black (%)	36 (11.84%)	
Other (%)	60 (19.74%)	
**CHRT-SR_9_**		
Total (range: 0–36)	15.69	8.14
Pessimism (range: 0–8)	4.45	2.19
Helplessness (range: 0–8)	4.47	2.38
Despair (range: 0–8)	3.47	2.41
Suicidal thoughts (range: 0–12)	3.30	3.09
PHQ-9 total (range: 0–27)	15.65	6.77

### Confirmatory factor analysis

We fit CHRT-SR_9_ to the data (Figure 1). The Bollen–Stine bootstrap *p* = 0.16 indicated good model fit. Model fit statistics such as CMIN/DF = 1.54, CFI = 0.995, TLI = 0.991, and RMSEA = 0.038 also indicated excellent model fit to the data. Means and SDs for the subfactor and total scores can be found for the overall sample at first and second visits in Tables 1, 2.

### Evaluation of invariance by sex

We checked model fit for males and females (Supplementary Figure 1); both fit well on all metrics. Configural invariance (CMIN/DF = 1.44, CFI = 0.992, TLI = 0.985, and RMSEA = 0.035), metric invariance ($\chi_{5}^{2}=6.19;$ *p*-value = 0.29) and scalar invariance ($\chi_{5}^{2}=6.04;$ *p*-value = 0.30) were upheld, suggesting that full scalar invariance held.

### Evaluation of invariance by age-groups

Model fit for the three age-groups: Young, middle aged, and older (Supplementary Figure 1) was excellent. Configural invariance (CMIN/DF = 1.36, CFI = 0.99, TLI = 0.98, and RMSEA = 0.03), metric invariance ($\chi_{10}^{2}=9.25;$ *p*-value = 0.51) and scalar invariance ($\chi_{2}^{2}=4.99;$ *p*-value = 0.08) were upheld.

### Classical test theory findings

The Spearman–Brown coefficient was calculated as a measure of internal consistency for each subscale and the total score. These reliability coefficients indicated excellent reliability for the total score and all subfactors at both first (0.76–0.90) and second visit (0.81–0.92) (Table 2). Additionally, we calculated the corrected item-total correlation for each item at the first visit and these varied between 0.57 and 0.79 (Table 3).

**TABLE 2:** Internal consistency reliability and sensitivity to change between visits.

First visit (*n* = 369)			Second visit (*n* = 369)		
Measure	Mean ± SD	Spearman–Brown coefficient	Mean ± SD	Spearman–Brown coefficient	*P*-value
Pessimism (items 1 and 2)	4.45 ± 2.19	0.76	4.06 ± 2.33	0.85	0.02
Hopelessness (items 3 and 4)	4.47 ± 2.38	0.90	4.24 ± 2.37	0.92	0.19
Despair (items 8 and 9)	3.47 ± 2.41	0.84	3.05 ± 2.28	0.86	0.01
Suicidal thoughts (items 12, 13, and 14)	3.30 ± 3.09	0.83	2.82 ± 2.72	0.83	0.02
Total score	15.69 ± 8.14	0.78	14.16 ± 8.12	0.81	0.01

**TABLE 3:** CHRT-SR_9_ item frequencies and item total correlation at first visit.

CHRT-SR_9_ items	Mean	SD	Corrected item total correlation
**Pessimism**			
CHRTSR_01: I feel as if things are never going to get better.	2.50	1.19	0.60
CHRTSR_02: I have no future.	1.95	1.25	0.70
**Hopelessness**			
CHRTSR_03: It seems as if I can do nothing right.	2.32	1.24	0.66
CHRTSR_04: Everything I do turns out wrong.	2.14	1.25	0.66
**Despair**			
CHRTSR_08: I feel that there is no reason to live.	1.55	1.23	0.79
CHRTSR_09: I wish I could just go to sleep and not wake up.	1.91	1.36	0.68
**Suicidal thoughts**			
CHRTSR_12: I have been having thoughts of killing myself.	1.37	1.27	0.67
CHRTSR_13: I have thoughts about how I might kill myself.	1.22	1.22	0.65
CHRTSR_14: I have a plan to kill myself.	0.71	0.90	0.57

### Sensitivity to change

In total, 369 adults completed both first and second visit CHRT-SR_9_ measurements. Although the length of time between visits varied by individual, all second visits occurred within 4 months of the first (mean time to second visit = 40.7 days, median = 30 days, max = 120 days). Table 2 shows that CHRT-SR_9_ scores were sensitive to change following the first visit, with the average subfactor and total scores decreasing significantly by the second visit.

### Performance of CHRT-SR_9_ against item 9 of PHQ-9

The cross-sectional and change analyses allowed us to estimate the relative current risk, as measured by the CHRT-SR_9_, against the single suicide item in PHQ-9. All participants completed the PHQ-9 questionnaires at both visits. At the first visit, 31.3% (*n* = 115) adults indicated “Not at all” to Item 9, 39.0% (*n* = 143) “several days,” 18.8% (*n* = 69) “More than half the days,” and 10.9% (*n* = 40) “Nearly every day.” Table 4 shows the means/SDs for each item, as well as for the subscale scores, across the response categories of Item 9. The CHRT-SR_9_ means were significantly higher with greater levels of suicidality as reflected in responses to Item 9. Taking the total score in CHRT-SR_9_ as the overall measure of suicidality and comparing it to the levels of Item 9, we found that no risk (0 on Item 9) corresponded to a mean total score on CHRT-SR_9_ of 7.82 (*SD* = 5.53), mild (1 on Item 9) was 16.80 (*SD* = 4.99) on CHRT-SR_9_, moderate was 20.71 (*SD* = 5.36) and severe was 25.95 (*SD* = 7.30).

**TABLE 4:** Means of CHRT item/subscale scores for each response to the PHQ-9 suicide item at 1st visit.

PHQ-9 item 9 responses					
Numbers responding	0 (*n* = 115)	1 (*n* = 143)	2 (*n* = 69)	3 (*n* = 40)	
CHRT items	Mean (SD)	Mean (SD)	Mean (SD)	Mean (SD)	*P*-value
CHRTSR_01: I feel as if things are never going to get better.	1.66 (1.16)	2.69 (1.01)	2.99 (0.90)	3.38 (0.90)	<0.0001
CHRTSR_02: I have no future.	1.10 (1.08)	2.08 (1.04)	2.49 (1.11)	3.05 (1.18)	<0.0001
CHRTSR_03: It seems as if I can do nothing right.	1.54 (1.26)	2.47 (0.96)	2.91 (1.03)	3.13 (1.20)	<0.0001
CHRTSR_04: Everything I do turns out wrong.	1.36 (1.18)	2.26 (1.03)	2.75 (1.05)	3.00 (1.28)	<0.0001
CHRTSR_08: I feel that there is no reason to live.	0.58 (0.81)	1.61 (0.93)	2.23 (1.06)	3.00 (1.26)	<0.0001
CHRTSR_09: I wish I could just go to sleep and not wake up.	0.83 (1.01)	2.02 (1.08)	2.70 (1.13)	3.35 (1.12)	<0.0001
CHRTSR_12: I have been having thoughts of killing myself.	0.30 (0.55)	1.59 (1.07)	1.96 (1.19)	2.78 (1.19)	<0.0001
CHRTSR_13: I have thoughts about how I might kill myself.	0.27 (0.57)	1.37 (1.07)	1.68 (1.19)	2.60 (1.19)	<0.0001
CHRTSR_14: I have a plan to kill myself.	0.18 (0.41)	0.72 (0.82)	1.00 (0.91)	1.68 (1.14)	<0.0001
**CHRT-SR_9_ subscales**					
Pessimism (range: 0–8)	2.77 (1.96)	4.77 (1.71)	5.48 (1.75)	6.43 (1.85)	<0.0001
Helplessness (range: 0–8)	2.90 (2.35)	4.73 (1.79)	5.67 (1.95)	6.13 (2.33)	<0.0001
Despair (range: 0–8)	1.41 (1.61)	3.63 (1.75)	4.93 (1.89)	6.35 (2.26)	<0.0001
Suicidal thoughts (range: 0–12)	0.75 (1.34)	3.68 (2.52)	4.64 (2.89)	7.05 (3.04)	<0.0001
**Total score** (range: 0–36)	7.82 (5.53)	16.80 (4.99)	20.71 (5.36)	25.95 (7.30)	<0.0001

### Does CHRT-SR_9_ change over time with a change in suicidality?

A meaningful change in risk can be estimated by the degree of change in PHQ-9 Item 9 responses. Table 5 lists the mean change in each item, as well as total and subscale scores of the CHRT-SR_9_, against change in Item 9, from the first visit to the second visit. Lower change scores implied improvement at second visit compared to first visit while higher values implied worsening. As the PHQ-9 Item 9 progressed from improvement to worsening, CHRT-SR_9_ items moved similarly from improvement to worsening. For example, on average, (i) a 3-point improvement in Item 9 (from 3 at first visit to 0 at second visit) corresponded to 13 points (*SD* = 7.48) drop in CHRT-SR_9_ total score, (ii) a 2-point improvement in Item 9 (e.g., 3–1 or 2–0) corresponded to 7.23 points (*SD* = 8.16) drop in CHRT-SR_9_ total score, etc. (Table 5).

**TABLE 5:** Mean changes in CHRT item/subscale scores by change in PHQ-9 suicide item.

Changes in PHQ-9 suicide item from baseline to second visit (minus is improvement)						
	Improved			No change	Worsened	
	−3 (*n* = 5)	−2 (*n* = 26)	−1 (*n* = 72)	0 (*n* = 213)	1 (*n* = 38)	2 (*n* = 10)
Changes in CHRT-SR_9_: 2nd–1st visit	Mean (SD)	Mean (SD)	Mean (SD)	Mean (SD)	Mean (SD)	Mean (SD)
CHRTSR_01: I feel as if things are never going to get better.	−1.40 (1.52)	−1.19 (1.50)	−0.51 (1.21)	−0.17 (1.18)	0.37 (1.40)	0.70 (1.57)
CHRTSR_02: I have no future.	−1.20 (1.30)	−0.88 (1.31)	−0.39 (1.00)	−0.12 (0.96)	0.50 (1.16)	1.20 (1.40)
CHRTSR_03: It seems as if I can do nothing right.	−1.40 (1.14)	−0.54 (1.27)	−0.57 (1.12)	−0.08 (1.11)	0.66 (1.17)	0.90 (1.20)
CHRTSR_04: Everything I do turns out wrong.	−1.20 (1.30)	−0.69 (1.44)	−0.40 (0.96)	−0.08 (1.04)	0.42 (1.13)	1.30 (1.16)
CHRTSR_08: I feel that there is no reason to live.	−1.40 (1.52)	−0.85 (1.41)	−0.50 (0.86)	−0.15 (0.76)	0.16 (0.89)	1.10 (1.37)
CHRTSR_09: I wish I could just go to sleep and not wake up.	−2.20 (1.10)	−0.96 (1.37)	−0.58 (1.16)	−0.16 (1.07)	0.63 (1.40)	1.20 (1.40)
CHRTSR_12: I have been having thoughts of killing myself.	−2.00 (1.41)	−0.92 (1.52)	−0.74 (1.17)	−0.08 (0.85)	0.42 (1.22)	1.40 (1.26)
CHRTSR_13: I have thoughts about how I might kill myself.	−2.00 (1.41)	−0.69 (1.52)	−0.61 (1.15)	−0.16 (0.79)	0.45 (1.03)	1.40 (0.84)
CHRTSR_14: I have a plan to kill myself.	−0.20 (0.84)	−0.50 (1.10)	−0.29 (0.86)	−0.01 (0.63)	0.18 (0.87)	0.20 (0.92)
**Subscales**						
Pessimism	−2.60 (2.61)	−2.08 (2.51)	−0.90 (1.90)	−0.29 (1.79)	0.87 (2.35)	1.90 (2.64)
Helplessness	−2.60 (2.30)	−1.23 (2.37)	−0.97 (1.79)	−0.16 (1.93)	1.08 (2.06)	2.20 (2.30)
Despair	−3.60 (2.51)	−1.81 (2.45)	−1.08 (1.57)	−0.31 (1.57)	0.79 (1.91)	2.30 (2.63)
Suicidal thoughts	−4.20 (2.77)	−2.12 (3.77)	−1.64 (2.74)	−0.25 (1.80)	1.05 (2.22)	3.00 (2.36)
Total score	−13.00 (7.48)	−7.23 (8.16)	−4.60 (5.26)	−1.02 (4.73)	3.79 (6.51)	9.40 (8.36)

## Discussion

In a large sample of adults seen in primary care practices, the brief, 9-item version of the CHRT-SR was identified and evaluated. These nine known items were identical to those identified by similar methods in a representative sample of adolescent outpatients (9). In addition, the performance of the total scale and the subscales were highly comparable to results found in the adolescent population.

The four factors or subscales (pessimism, helplessness, despair, and suicidal thoughts) have clinical face validity, as each construct has been associated with suicidal risk in many studies over the years. For example, pessimism (“I feel as if things are never going to get better” and “I have no future”) is well known to be associated with suicidal risk (18). The helplessness subscale includes responses to “It seems as if I can do nothing right” and “Everything I do turns out wrong,” which certainly reflects a sense of inefficacy, which also is associated with suicidal risk (19). The third subscale we call despair, as “I feel that there is no reason to live” and “I wish I could just go to sleep and not wake up” reflects a resignation to fate and a sense that struggling does not matter, which also is often found in suicidal notes (19). Finally, the 3-item suicidal thinking/planning subscale (“I have been having thoughts of killing myself;” “I have thoughts about how I might kill myself;” “I have a plan to kill myself”) would be expected to relate to the propensity to end things. In addition, the three 2-item subscales on the CHRT-SR_9_ (pessimism, helplessness, and despair) are distinct and have clinical face validity.

Of note, is the lower corrected item-total correlation for item 14 (“I have a plan to kill myself”). This is likely because this is a general population not seeking care for suicidal thoughts, and not restricted to depressed patients (although there were depressed adults included in the sample). Thus, the prevalence of patients with a suicide plan would be low and thereby reduce the item-total correlation.

Establishment of measurement invariance by gender and age groups indicated that mean differences among sub-groups were real and not attributable to measurement bias, making comparisons of CHRT-SR_9_ total and subscale scores between males and females or among different age groups valid. A granular look at suicidality by gender and age, through the lens of CHRT-SR_9_, is of significant interest and deserves further study.

Overall, the CTT and the concurrent validity analyses revealed a coherent, internally consistent instrument that can detect both the improvement and worsening over time in this population. The cross-sectional and change analyses allow us to estimate the relative current risk as measured by the CHRT-SR_9_ against the single PHQ-9 suicide Item 9. It gives us estimates of CHRT-SR_9_ total scores that correspond to *no, mild, moderate*, and *severe risk* on Item 9. It also gives us estimates of *changes* in CHRT-SR_9_ total score corresponding to changes in Item 9 score between visits.

Prior reports on the psychometric properties of the CHRT-SR have largely been in adult samples acquired in the conduct of clinical trials, with the exception of our report in adolescents who were enrolled in a tertiary care suicide prevention program (9). There have been attempts to explore a variety of versions of the CHRT-SR to facilitate implementation in practice and research studies, with different versions allowing for assessment of specific symptoms (i.e., impulsivity and irritability), as well as shortening of the scale. These versions include the 16, 14, 12, and the 7-item CHRT-SR (8, 20–27). These versions of the CHRT-SR have been found to have acceptable psychometric properties. It would, therefore, not be surprising that a limited number (9) of items in adults have similar desirable properties.

A briefer tool with acceptable psychometric features, which is demonstrated to be applicable to both adolescents and adults, makes identification of suicidality in a busy primary care practices less demanding and more feasible, especially if a self-report is used. Secondly, nine items are few enough to be adaptable to digital administration *via* smartphones or otherwise, thus offering a therapeutic opportunity for early and perhaps targeted intervention. Thirdly, this 9-item scale with four clinically relevant subscales, can help clinicians identify and focus their discussion on factors that are particularly contributing to the risk of suicidality. This report is an initial attempt to establish the degree of suicidal risk benchmarking the total score and subscale scores against the four possible responses to the PHQ-9 suicide Item 9.

This report has several limitations. The generalizability of findings to other primary care practices as well as public and private sector psychiatric practices is unknown. Concurrent validity based on other accepted measures of suicidality such as the Columbia-Suicide Severity Rating Scale (CSSRS), (28) and the Sheehan Suicidality Tracking Scale (Sheehan-STS) (29) is unknown. The degree to which the CHRT-SR_9_ predicts actual suicidal attempts or efforts to prevent such attempts in adolescents, as well as adults, is not known. Finally, its clinical utility as compared to clinical impression alone has not been assessed.

## Conclusion

In conclusion, the CHRT-SR_9_ is a brief self-report with excellent psychometric properties in both adolescents (based on our prior report) and adults (based on this report) that can estimate the degree of suicidality and whether a clinically important degree of improvement or worsening in suicidality has occurred. Its subscales provide clinical clues about psychological factors contributing to the risk.

## Data availability statement

The raw data supporting the conclusions of this article will be made available by the authors, without undue reservation.

## Ethics statement

The studies involving human participants were reviewed and approved by the UT Southwestern Medical Center Institutional Review Board. Written informed consent for participation was not required for this study in accordance with the national legislation and the institutional requirements.

## Author contributions

KN, AR, and MT conceptualized the study. KN and AR reviewed the literature, interpreted the results from the data analysis, and drafted the manuscript. KN analyzed the data. KN, AR, TC, TM, and MT revised the manuscript for important intellectual content. AR and MT provided project oversight. All authors approved the final version of the manuscript and take responsibility for the content herein.

## Licensing

Licensing and distribution of the CHRT-SR is managed by Mapi Research Trust on behalf of the copyright holder, University of Texas Southwestern Medical Center. At the time of publishing, the CHRT-SR is available without charge to non-commercial users. Fees may apply to commercial users, IT companies, funded academic users or healthcare organizations. Requests for information and licensing of the CHRT-SR should be submitted through Mapi Research Trust’s ePROVIDE platform (https://eprovide.mapi-trust.org/).
