# Supplementary figures and images for: The 9-item Concise Health Risk Tracking – Self-Report (CHRT-SR9) measure of suicidal risk: Performance in adult primary care patients
Source: Front Psychiatry. 2023 Feb 14;14:1014766. doi: 10.3389/fpsyt.2023.1014766 (PMC9971953; doi:10.3389/fpsyt.2023.1014766)

**Supplemental Figure 1.** CFA for CHRT-SR<sub>9</sub> by Gender and Age groups

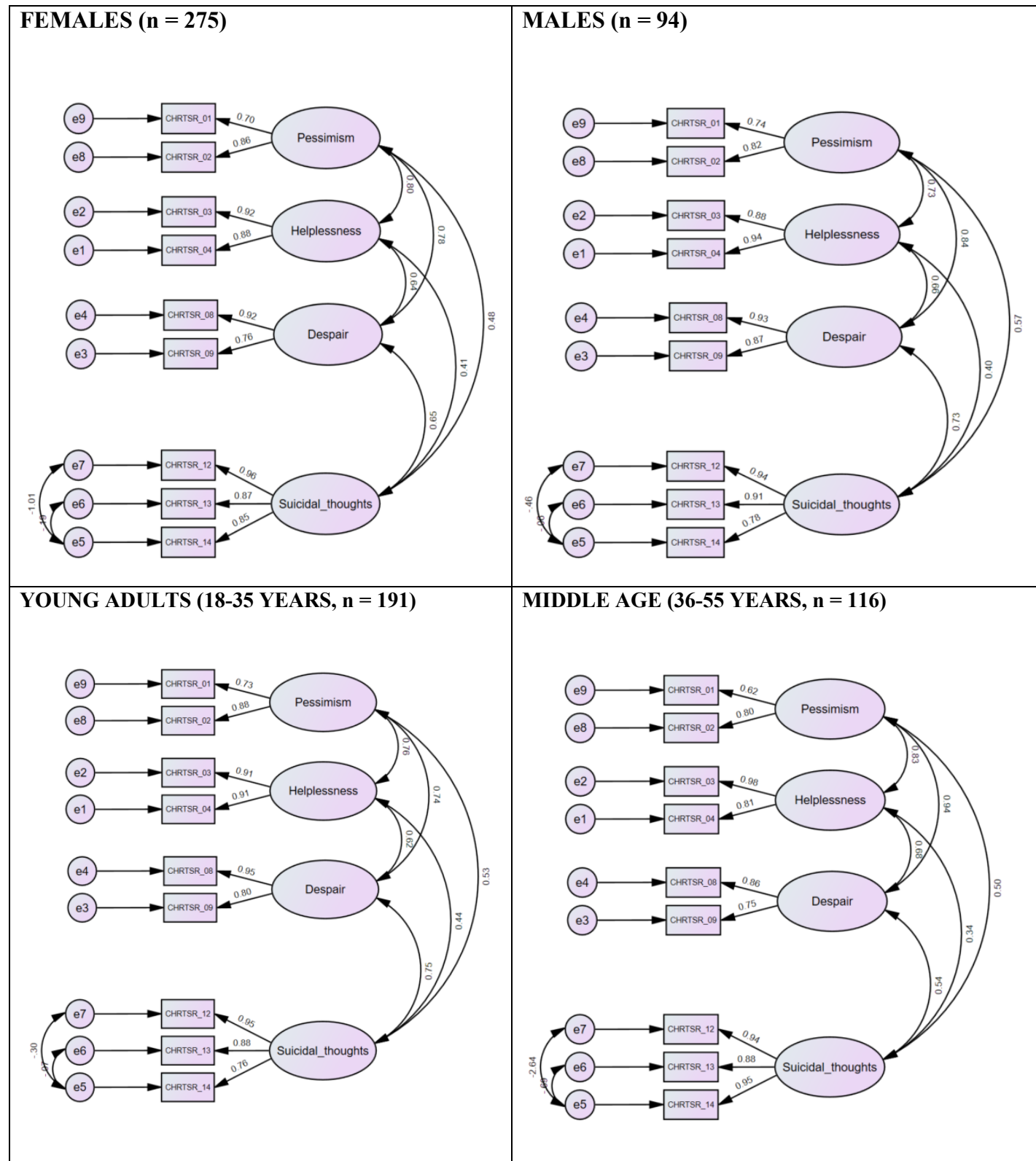

**OLDER ADULTS (>55 YEARS, n = 62)**

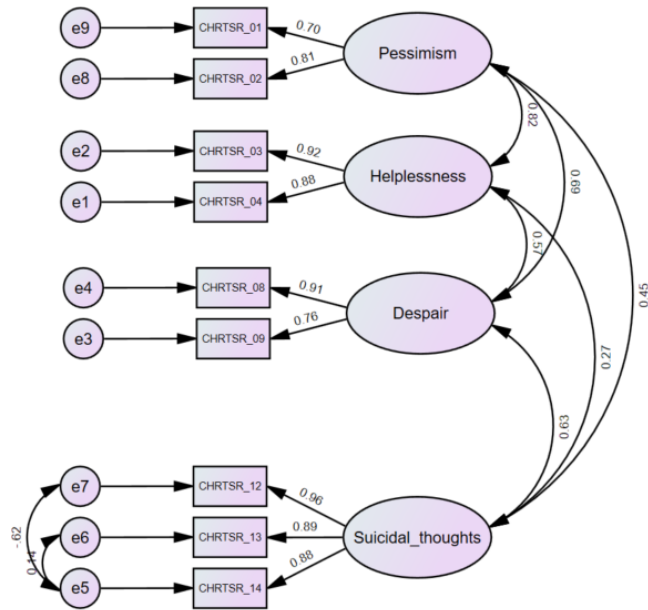

Supplement: Supplementary file 1 [file Data_Sheet_1.PDF]
